# Doctors Disagree

**Published:** 1892-04

**Authors:** Ella A. Jennings

**Affiliations:** Editor Humanity and Health; Humanity Publishing Company, 93 Clinton Place, New York


### DOCTORS DISAGREE.

Humanity Publishing Company, 93 Clinton Place, New York.

To the Editor of Hall's Journal of Health :

Dear Sir :—Under the caption of "Women and their Duty," in your March number, is a statement by Dr. Arabella Kenealy, a lady physician of London, England, to which I would like to reply, knowing that the experiences of others is helpful to each of us.

Her experience has been the opposite of mine. In fifteen years of constant medical practice I have observed that children born of mothers who led an active life, both *mentally and physically*, *were far superior to those* whose mothers led an inactive or quiescent life. This was especially true in the children of women who were engaged in professions and other mental activities or avocations. I found them much finer, stronger and more intelligent than those children born in the upper classes, where comfortable idleness prevailed, or among the lower classes, where poverty and idleness were often coupled. The middle classes are our main reliance, and they recruit our broken ranks.

I have been a student of nature all my life, and have had a large obstetrical experience, having been present at over three hundred births. I have attended other physicians in their accouchements, and recall to mind that of one whom, of all overworked women, I never saw her equal. She did the work of four average persons from 3 and 4 A. M. till 10 and 12 P. M. She was ever busy. She told me that she saw from 40 to 70 patients, office and visiting, in a day, attended to her household duties, and walked six to eight miles daily from choice, and that she had been unusually severely taxed during her period of gestation. Yet this child was the finest she had had, and, in fact, superior to any child I have ever seen. At six weeks she could sit up in arms; at four months had five teeth; at ten months walked all over, and could go up and down stairs with safety. She had not cried six hours in her daily life, nor kept any one awake nights, nor had colic, but was altogether the best baby this mother and physician had had. Later on the child's ability and strength was phenomenal. She could walk four to six miles, and never complained of fatigue, but was always like a sunbeam, and had superior mental as well as physical powers. This mother told me she had proven many theories in regard to heredity that she had entertained for years through her experience with this child. That this baby was a reproduction of her various exalted states, and a proof of what loving maternity uninvaded could accomplish. This child was not a chance one, but the result of a most intense desire for a child, and was a culmination of the highest in that mother that motherhood could impart.

For over twenty-five years I have been studying the conditions that will improve animals and human beings, and I know by observation of facts, and professional and personal experience, that we can produce the kind of children we desire, provided we are willing to live the kind of life necessary to ensure the favorable conditions. I have tested this matter in many cases professionally, and received the confidences of hundreds of women upon the subject, and found that the best children resulted from those mothers who led an active professional life.

ELLA A. JENNINGS, M. D.,
Editor Humanity and Health.

